# Supplementary material for: The Farmed Atlantic Salmon (Salmo salar) Skin–Mucus Proteome and Its Nutrient Potential for the Resident Bacterial Community
Source: Genes (Basel). 2019 Jul 7;10(7):515. doi: 10.3390/genes10070515 (PMC6678340; doi:10.3390/genes10070515)
Supplement: Supplementary file 1 [file genes-10-00515-s001.zip › genes-505484-figure.docx]

Supplementary figures

**The farmed Atlantic salmon (*Salmo salar*) skin-mucus proteome and its nutrient potential for the resident bacterial community**

Giusi Minniti^1^, Simen Rød Sandve^2^, János Tamás Padra^3^, Live Heldal Hagen^1^, Sara Lindén^3^, Phillip B. Pope^1^, Magnus Ø. Arntzen^1*^ and Gustav Vaaje-Kolstad^1*^

^1^Faculty of Chemistry, Biotechnology and Food Science, Norwegian University of Life Sciences (NMBU), Ås, Norway

^2^Faculty of Biosciences, Norwegian University of Life Sciences (NMBU), Ås, Norway.

^3^Department of Medical Biochemistry and Cell Biology, Sahlgrenska Academy, University of Gothenburg, Gothenburg, Sweden.

*Corresponding authors: Gustav Vaaje-Kolstad & Magnus Ø. Arntzen


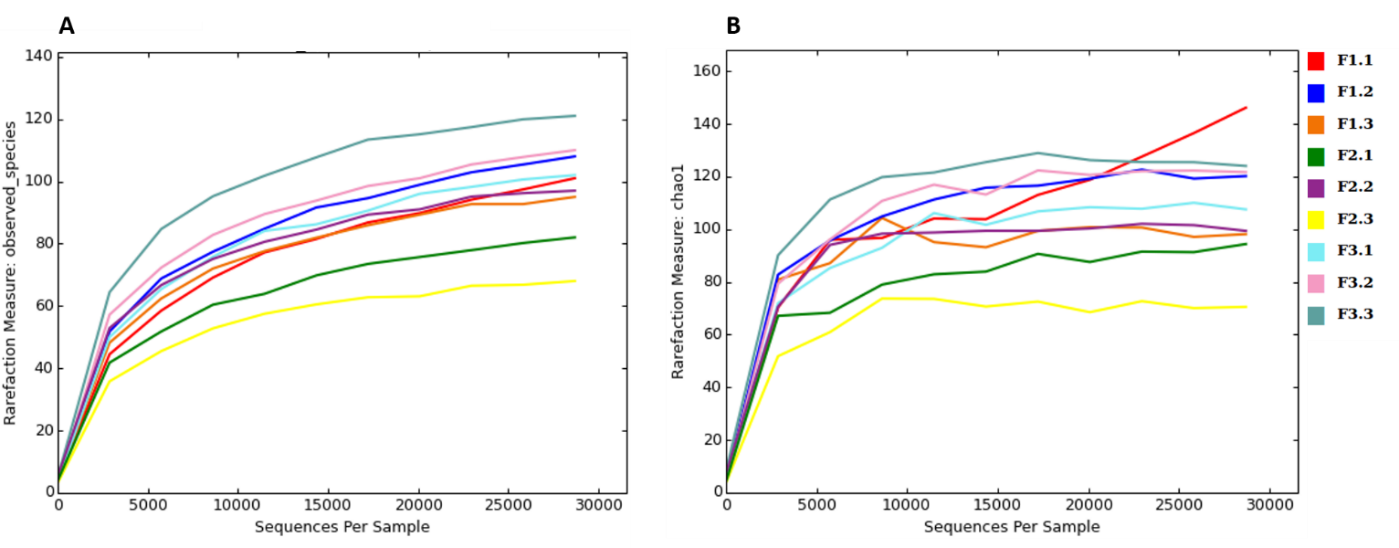


**Figure S1**: Alpha diversity rarefaction curves of each sample, presented as the number of observed species (OTUs) in Panel A and Chao1 in Panel B. Note that the sequencing depth was normalized to the sample with lowest number of sequences (n = 28.893) and singletons were discarded prior alpha diversity analysis.

**
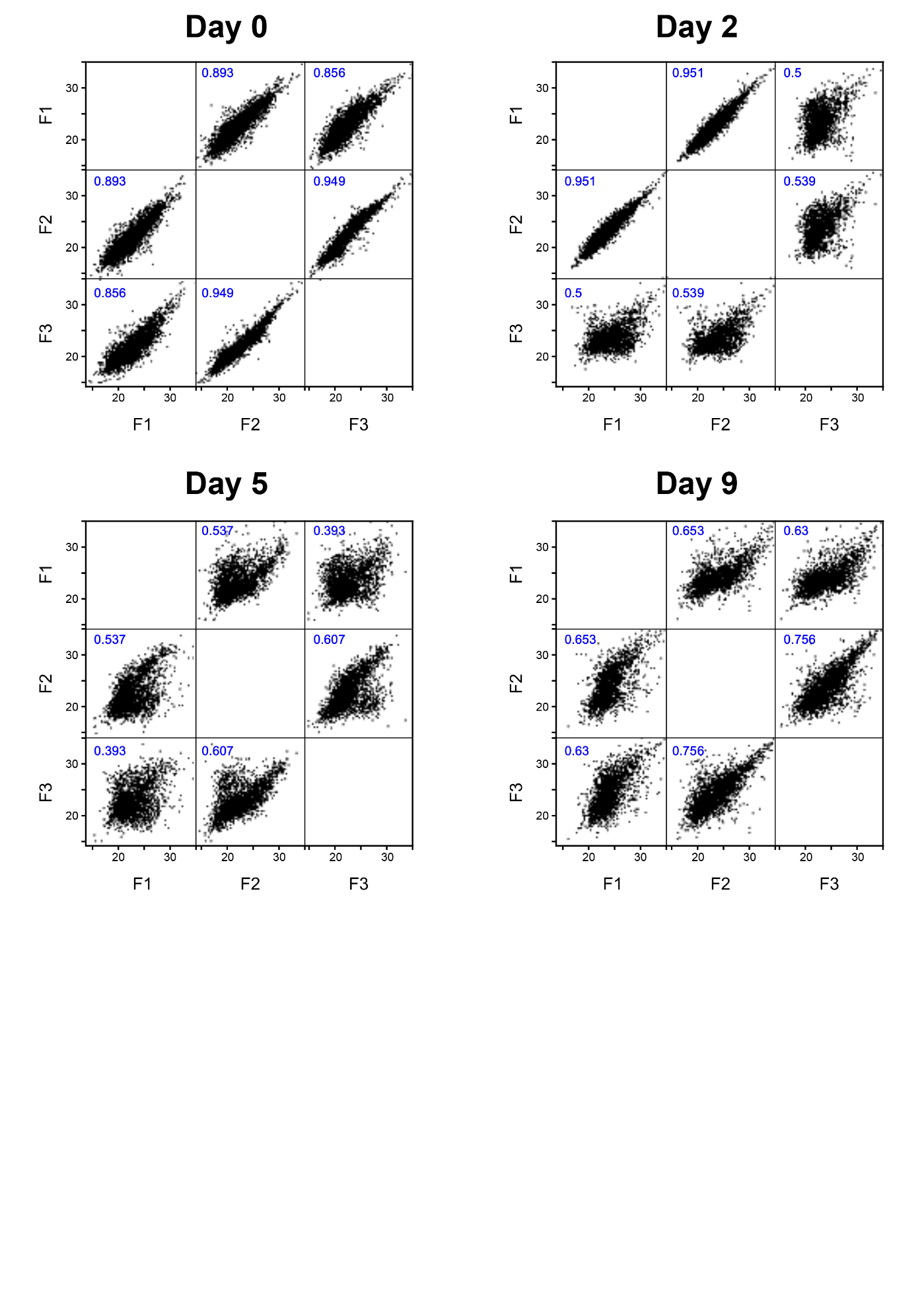
**

**Figure S2.** Scatterplot matrix to visualize replicate consistency of mucus samples (mucus from three fish; F1, F2 and F3) incubated with sterialized seawater and harvested at Day 0, 2, 5 and 9. X- and Y-axis indicate log2-transformed protein abundance and the numbers inside the plots indicate Pearson correlation, ranging between R=0.39-0.95.


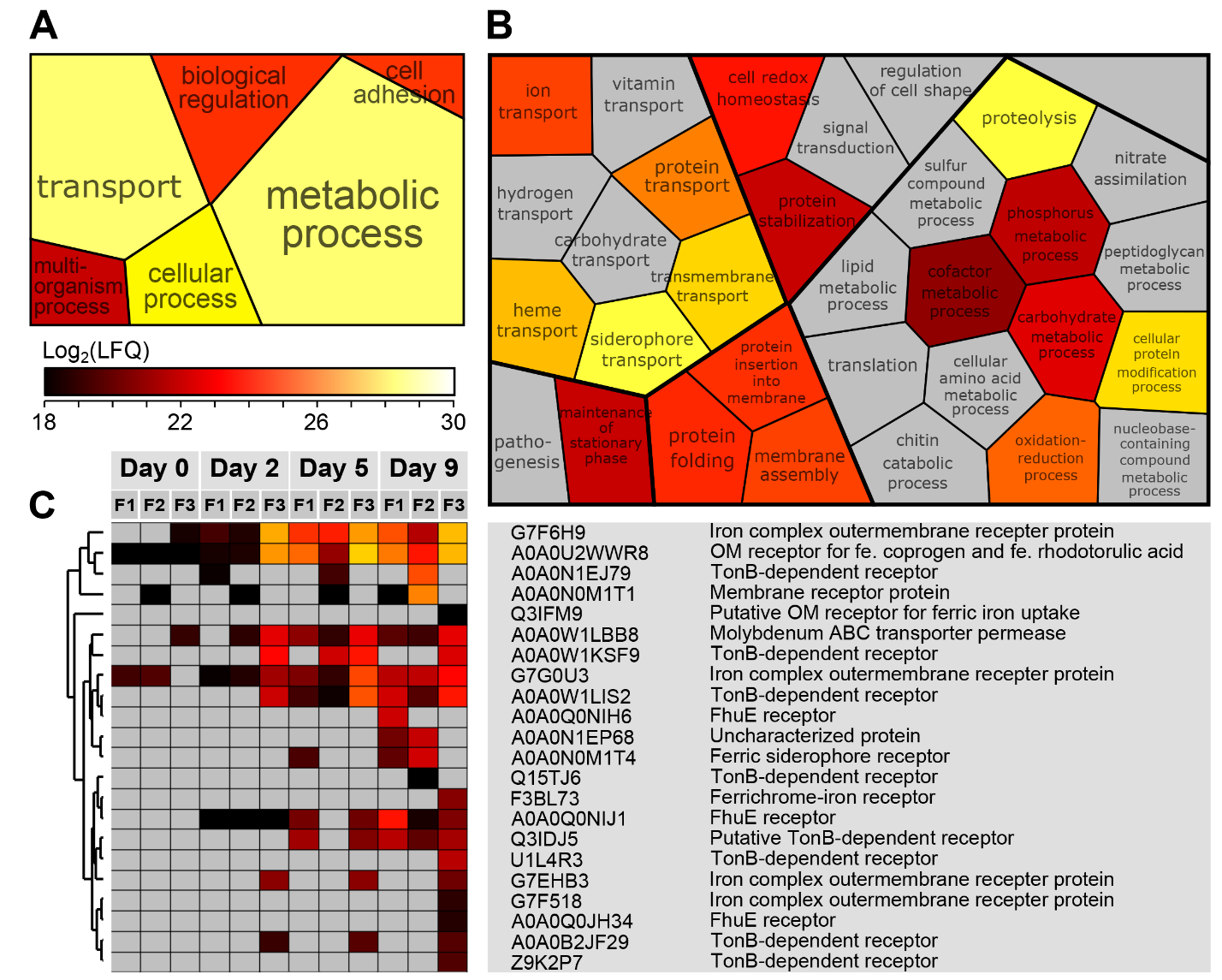


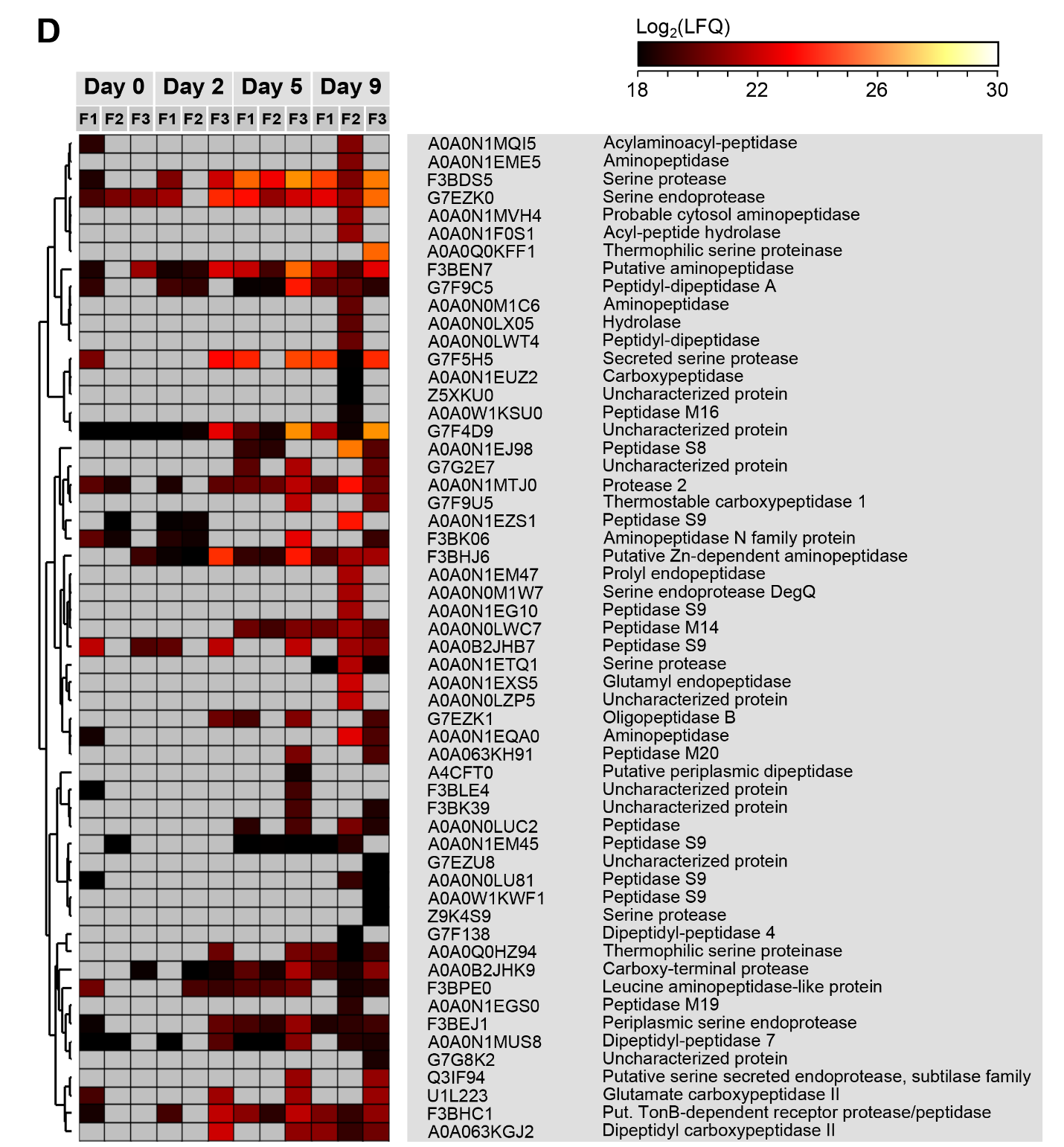


**Figure S3.** ***Pseudoalteromonas* secreted proteins assigned into different groups according to their biological functions**. Panel (A) shows a set of high-level GO terms where the size of each block indicate the number of child GO-terms as depicted in B, and the color of each block reflects the abundance. Panel (B) shows a similar representation for medium-level GO terms as children of the blocks in A. Grey blocks indicate that no proteins could be mapped to this GO-term. Panel (C) shows a heat map of the proteins associated with ‘siderophore transport’, while Panel (D) shows proteins assigned to ‘proteolysis’. The heat map ranges from low abundance (black), to medium abundance (red) and high abundance (white).


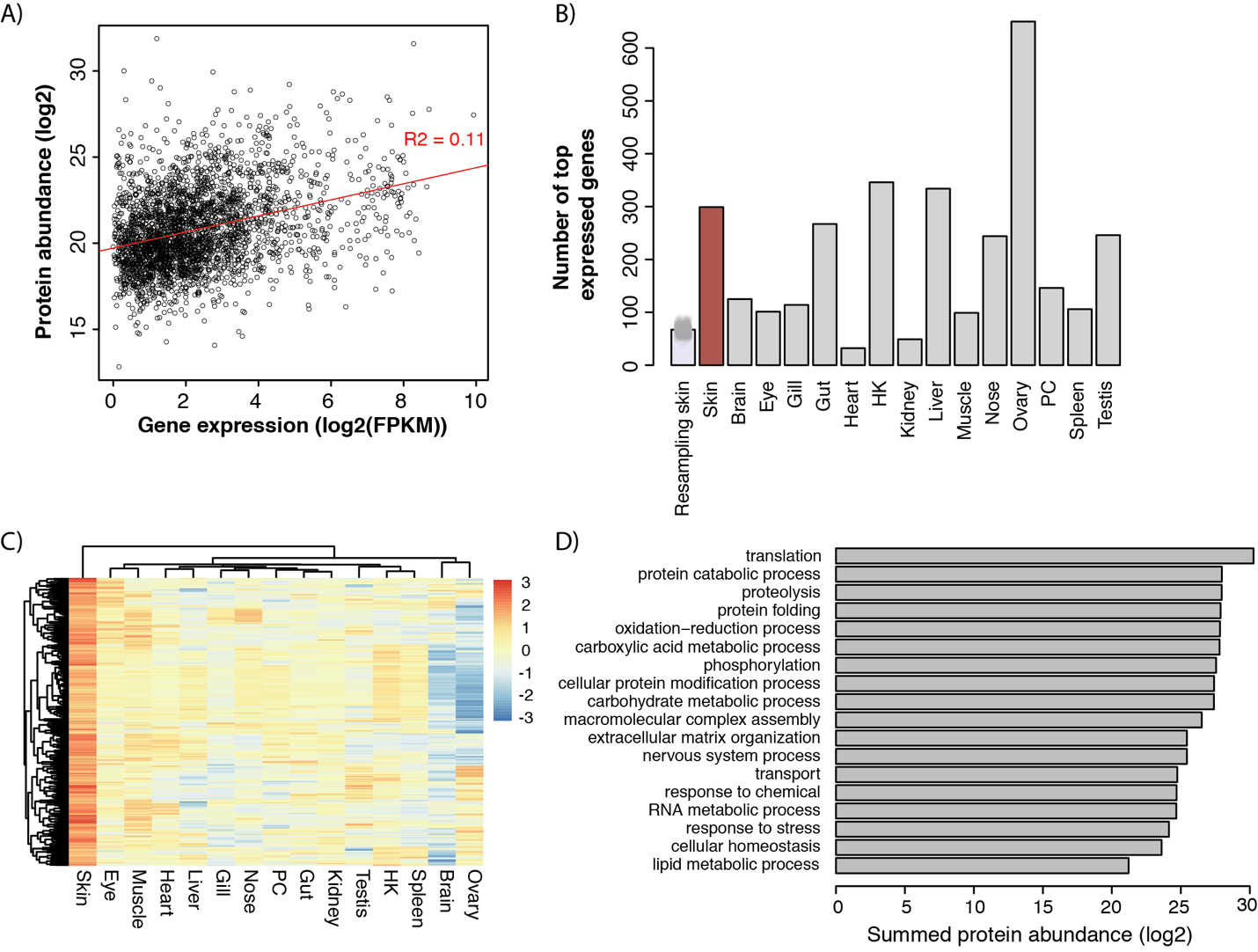
**Figure S4.** **Tissue gene expression**. **A)** Correlation between skin surface protein abundance and gene expression of the same proteins in skin tissue. The positive correlation is highly significant in a pearson correlation test (p=4.59^-75^). A linear model fitted to the data and the proportion of the variance explained is indicated with red line and text. **B)** Number of genes with the highest expression across all tissues in the skin protein gene set. Of the 3158 genes in the gene set, 299 had the highest expression in skin. The “resampling skin” bar shows the mean (bar height) and distribution (grey points) of the number of genes with highest expression in skin in 1000 random samples of 3158 protein coding genes. **C)** Heatmap showing the tissue expression distribution for the 299 genes with highest expression in skin. Expression levels are scaled by rows. Red indicates higher expression and blue lower expression. **D**) The most prevalent biological processes (GO) and their summed protein abundance of the 299 proteins with highest expression in skin.
